# Autophagy in plants and algae

**DOI:** 10.3389/fpls.2014.00679

**Published:** 2014-12-01

**Authors:** Diane C. Bassham, Jose L. Crespo

**Affiliations:** ^1^Department of Genetics, Development and Cell Biology, Iowa State UniversityAmes, IA, USA; ^2^Instituto de Bioquímica Vegetal y Fotosíntesis, Consejo Superior de Investigaciones Científicas-Universidad de SevillaSeville, Spain

**Keywords:** selective autophagy, lipid degradation, plants, algae, pexophagy

Autophagy is a major cellular degradation pathway in which materials are delivered to the vacuole in double-membrane vesicles known as autophagosomes, broken down, and recycled (Li and Vierstra, 2012; Liu and Bassham, 2012). In photosynthetic organisms, the pathway is strongly activated by biotic and abiotic stresses, including nutrient limitation, oxidative, salt and drought stress and pathogen infection, and during senescence (Perez-Perez et al., 2012; Lv et al., 2014). Mutation of genes required for autophagy causes hypersensitivity to stress, indicating that autophagy is important for tolerance of multiple stresses. While autophagy is often non-selective, a growing number of examples of selectivity are now evident, in which specific cargos are recruited into autophagosomes via cargo receptors (Floyd et al., 2012; Li and Vierstra, 2012). In this Research Topic, a series of original research articles and reviews highlight areas of current focus in plant and algal autophagy research, including mechanisms and cargos of selective autophagy, lipid degradation, and metabolic and physiological consequences of the autophagy pathway.

Several contributions to the Research Topic address the emerging concept of selective autophagy, well established in animal cells but only described recently in plants. Zientara-Rytter and Sirko (2014) in a research article follow up on previous work describing a potential selective autophagy receptor in tobacco, Joka2, identified as possibly functioning in responses to sulfur deficiency (Zientara-Rytter et al., 2011). They perform a functional analysis of protein domains within Joka2, identifying domains responsible for homodimerization and for the sequestration of cargo, tagged with ubiquitin, into aggregates within the cytoplasm. Zhou et al. (2014) address a potential function of the tomato Joka2 homolog, NBR1, in heat stress. They demonstrate that heat stress in tomato leads to activation of autophagy and that silencing of the core autophagy machinery, or of NBR1, leads to hypersensitivity to heat stress. In addition, silencing of tomato WRKY33 transcription factors causes heat sensitivity and reduced autophagy, suggesting that WRKY33 proteins are involved in the regulation of autophagy under these conditions. A likely cargo for Joka2/NBR1 is cytoplasmic protein aggregates, and Tasaki et al. (2014) describe a novel method for monitoring protein aggregate turnover by autophagy. They generate a fusion protein, Cyt b5-KikGR, which forms cytoplasmic aggregates and contains a photoconvertible fluorescent protein. Upon starvation of tobacco suspension cells for sucrose, phosphate, or nitrogen, the fluorescence is seen inside the vacuole after transfer of the aggregates by autophagy. Illumination of the aggregates with purple light converts the green fluorescence to red, enabling the authors to track autophagic transport of pre-existing vs. newly synthesized protein, thus allowing an assessment of autophagic flux.

Articles also discuss the selective autophagy of cellular organelles. Lee et al. (2014) review recent progress in the understanding of plant pexophagy, the selective degradation of peroxisomes by autophagy. Peroxisomal proteins are degraded in the developmental transition from glyoxysomes in seedlings to leaf peroxisomes and also as a quality control mechanism. Several groups have now demonstrated that this occurs by autophagy. The pathway for degradation of peroxisomes in tobacco suspension cells, both during sucrose starvation and under normal growth conditions, is described in a research article by Voitsekhovskaja et al. (2014). They demonstrate that peroxisomes are degraded in the vacuole by a mechanism that is sensitive to the autophagy inhibitor 3-methyladenine, suggesting a pexophagy pathway. Oku et al. (2014) describe interesting recent examples of pexophagy in plant-associated microorganisms, including a phytopathogenic fungus in which pexophagy is required for infection and a methylotrophic yeast residing on plant leaves, in which pexophagy is required for growth. Michaeli et al. (2014) review direct ER-to-vacuole transport pathways, including the transport of seed storage proteins and cysteine proteases by autophagy-related mechanisms. They discuss the recently identified ATI1 and 2 proteins, which bind to the autophagosome protein ATG8, are found in the endoplasmic reticulum under normal conditions, and are transported to the vacuole during starvation, features consistent with a selective autophagy mechanism. A review by Veljanovski and Batoko (2014) describes the potential selective autophagy of mitochondria, peroxisomes and endoplasmic reticulum.

Veljanovski and Batoko (2014) also discuss the mechanism by which individual proteins and other molecules can be degraded by autophagy. TSPO is a protein that can scavenge free heme, preventing its accumulation to toxic levels. TSPO binds to heme and causes its incorporation into autophagosomes, leading to vacuole delivery by autophagy. Another example that has recently come to light is the degradation of RNA silencing components by autophagy, discussed by Derrien and Genschik (2014). This pathway was originally discovered in the context of viral infection, but also occurs in mutants that are defective in RISC assembly, and possible physiological roles are discussed.

The degradation of lipid droplets by autophagy-related mechanisms is also a theme of the Research Topic. Zhao et al. (2014) study the transition from heterotrophic to autotrophic growth in the green microalga *Auxenochlorella protothecoides* in a research article. They analyze lipid droplet degradation and show that while macroautophagy is induced during the heterotrophic to autotrophic transition, lipid bodies are degraded in the vacuole by a microautophagy-like mechanism. Hanamata et al. (2014) discuss autophagy during male reproductive development in a review article. Rice autophagy mutants, unlike those of Arabidopsis, are male sterile, as pollen does not mature due to defects in the tapetum. Autophagy in tapetal cells is required for lipid body degradation, which in turn is involved in pollen maturation, highlighting an important difference between plant species. Lipophagy in phytopathogenic fungi is also discussed by Oku et al. (2014), as breakdown of lipid droplets is required for efficient infection but the mechanism is not well understood.

Finally, Ren et al. (2014) review the relationship of autophagy to carbon and nitrogen metabolism. Autophagy is known to function in starch degradation during the night and also to degrade chloroplast components during carbon deficiency. It is also involved in nitrogen remobilization from leaves during senescence, and autophagy mutants have lower nitrogen use efficiency. Transcriptome analysis indicates that several autophagy genes are found as hubs in transcriptional networks, an intriguing observation that should lead to interesting future experiments analyzing the role of these networks.

This collection highlights some of the recent advances in our understanding of plant autophagy and its role in numerous physiological processes and we hope that it will stimulate further discussion and research into this exciting topic.

## Conflict of interest statement

The authors declare that the research was conducted in the absence of any commercial or financial relationships that could be construed as a potential conflict of interest.
